# Cardiovascular Outcomes and Vascular Surrogate Markers in Rheumatoid Arthritis Patients Treated With Anti-Tumour Necrosis Factor (TNF) Therapy: A Systematic Review and Meta-Analysis

**DOI:** 10.7759/cureus.104389

**Published:** 2026-02-27

**Authors:** Danish Shah, Yashar Mashayekhi, Warda Batool Ali, Rajia Islam, Bismilla athar Dar, Syed Taqi Askari Shah, Eemahn Akhtar, Tooba Babar, Mohamed Anwar Mohamed Ibrahim, Elaf Mohamed Osman Ali Mohamed Salih, Husam Ali Abu Odeh

**Affiliations:** 1 Acute Medicine, Kettering General Hospital, England, GBR; 2 Trauma and Orthopaedics, University Hospitals of Leicester NHS Trust, Leicester, GBR; 3 Acute Medicine, Nottingham University Hospitals NHS Trust, Nottingham, GBR; 4 Stroke Medicine, Queens Hospital, England, GBR; 5 Internal Medicine, Quaid-e-Azam Medical College, Bahawalpur, PAK; 6 Trauma and Orthopaedics, Nottingham University Hospitals NHS Trust, Nottingham, GBR; 7 Medicine, Shifa International Hospital, Islamabad, PAK; 8 Internal Medicine, Combined Military Hospital, Rawlakot, PAK; 9 Medicine and Surgery, University of Gezira, Wad Madani, SDN; 10 General Practice, University of Gezira, Wad Madani, SDN; 11 General Practice, Ministry of Health Jordan, Amman, JOR

**Keywords:** anti-tnf therapy, cardiovascular risk, lipid profile, meta-analysis, rheumatoid arthritis, systematic review

## Abstract

Rheumatoid arthritis (RA) is linked with a high risk of cardiovascular disease (CVD), which is mainly triggered by chronic systemic inflammation. Tumour necrosis factor (TNF) inhibitors are effective in managing RA disease activity; however, their impact on cardiovascular risk markers is also unclear. The systematic review and meta-analysis aimed to assess the effects of anti-TNF therapy on conventional cardiovascular risk markers, specifically lipid parameters and blood pressure, in patients with rheumatoid arthritis. We searched PubMed, Embase, Google Scholar, and the Cochrane Library for randomised controlled trials published after 2010 that compared anti-TNF therapy with control treatments in adults with RA; 4 trials met the inclusion criteria. The lipid parameters (low-density lipoprotein cholesterol [LDL-C], high-density lipoprotein [HDL-C], triglycerides, and total cholesterol) and blood pressure were the outcomes. The Cochrane RoB 2.0 tool was used to measure risk of bias. Mean differences and 95% confidence intervals were used to produce random-effects meta-analyses. There were four randomised controlled trials with 295 participants. No statistically significant differences were found between the anti-TNF therapy and control groups for LDL-C, HDL-C, triglycerides, total cholesterol, systolic blood pressure, or diastolic blood pressure. The sensitivity analysis revealed that triglyceride results were unstable due to a single study, whereas the remaining results were very stable. In available RCTs, no statistically significant differences were observed between anti-TNF therapy and control treatments for conventional cardiovascular risk markers in patients with RA. More extensive and prolonged studies that include standardised cardiovascular outcomes should be undertaken to elucidate the cardiovascular effects of anti-TNF therapy.

## Introduction and background

Rheumatoid arthritis (RA) is a long-term autoimmune condition marked by inflammation of the synovial lining, gradual joint deterioration, and involvement of multiple organ systems [1]. Besides affecting the musculoskeletal system, RA has been linked to a significantly heightened risk of cardiovascular disease (CVD), which is one of the leading causes contributing to disease burden and death in this population [2]. Systemic inflammation in RA results in accelerated atherosclerosis, endothelial dysfunction, and altered vascular remodelling, underlying factors of the increased cardiovascular risk in these patients [3].

Tumour necrosis factor-alpha (TNF-alpha) is a key determinant in the pathophysiology of RA; it triggers synovial inflammation and systemic immune activation [4]. Consequently, TNF inhibitors (anti-TNF therapy) have become an essential part of RA treatment, successfully reducing disease activity and improving quality of life [5]. Recent cohort studies have also reported that RA patients undergoing anti-TNF treatment have low incidences of myocardial infarction and other cardiovascular events [6,7].

There is also emerging evidence of cardiovascular benefits associated with anti-TNF agents, which may act by regulating inflammatory pathways involved in atherogenesis and vascular pathology [8]. Surrogate measures of vascular health, including arterial stiffness, endothelial function, and carotid intima-media thickness, have also been improved in RA patients who received anti-TNF therapy, as reported by observational studies and randomised trials [9]. Although these results are positive, the effects of anti-TNF treatment on clinically significant cardiovascular outcomes remain incompletely understood. The inconsistent findings across studies and differences in study design, follow-up time, and endpoint outcomes require a thorough synthesis of the evidence [9,10]. Moreover, recent studies show that TNF inhibitors reduce inflammation-mediated cardiovascular risk in patients with RA, and ongoing studies are examining both surrogate markers and clinical outcomes to help clarify their role in long-term cardiovascular improvement [11].

Although previous studies suggest potential cardiovascular benefits of anti-TNF therapy, the randomised evidence remains limited, and outcomes are inconsistently reported across trials. Prior systematic reviews have often included observational studies or pooled heterogeneous cardiovascular endpoints, making it difficult to draw firm conclusions. Importantly, data from RCTs on clinical cardiovascular events and imaging-based vascular outcomes (e.g., carotid intima-media thickness, arterial stiffness, and endothelial function) remain insufficient for quantitative synthesis. Therefore, the present systematic review and meta-analysis focuses on conventional cardiovascular risk markers, specifically lipid parameters and blood pressure, as these were the most consistently reported outcomes in eligible post-2010 RCTs. This review aims to provide an updated pooled estimate of the effect of anti-TNF therapy on these cardiovascular risk-related biomarkers in patients with rheumatoid arthritis.

Objectives

The main goal of this systematic review and meta-analysis is to determine the effect of anti-TNF therapy on cardiovascular risk factors, specifically lipid profile and blood pressure, as surrogate indicators of vascular health, in patients with rheumatoid arthritis. This study focuses on these surrogate markers due to the limited availability of data on clinical cardiovascular events, such as myocardial infarction, stroke, and cardiovascular mortality. Combining findings from various studies, the work aims to determine whether anti-TNF therapy reduces systemic inflammation and translates into measurable improvements in cardiovascular risk parameters in RA patients.

## Review

Materials and methods

The research was conducted following the PRISMA (Preferred Reporting Items for Systematic Reviews and Meta-Analyses) principles, and the research protocol was pre-registered at PROSPERO (CRD420261285555) [12].

Search strategy

We systematically searched four databases for articles related to our search. We used specific keywords to search for articles and combined them using Boolean operators, i.e., 'AND' and 'OR'. A particular search strategy was used for each database. The databases we included were PubMed, Google Scholar, The Cochrane Library, and Embase. The keywords used to develop the search strategy were "rheumatoid arthritis", "anti-TNF therapy", "tumour necrosis factor inhibitors", "cardiovascular outcomes", "major adverse cardiovascular events", "endothelial function", and "vascular surrogate markers". We searched only for randomised controlled trials published after 2010. Additionally, references from the included papers and papers citing them were searched for inclusion in our study.

Selection criteria

We included only articles that reported participants aged ≥18 years diagnosed with rheumatoid arthritis. Studies needed to include one of the anti-TNF agents (e.g., adalimumab, infliximab, or etanercept) in the treatment group and a comparator, such as a placebo, conventional synthetic DMARDs, or non-TNF biologic therapies. Additionally, studies needed to report on at least one of the lipid markers to be included in the analysis. As mentioned above, only RCTs published after 2010 were included.

We excluded studies not published in English and studies that included patients other than those with rheumatoid arthritis (RA) or RA patients not receiving an anti-TNF agent in the treatment arm, as well as studies with patients with other autoimmune diseases that could lead to arthritis. Similarly, abstracts, reviews, cohorts, cross-sectional studies, and case reports were excluded. Studies that did not mention any of the lipid markers were also excluded.

Screening, selection, and data extraction

After retrieving articles from our selected databases, we searched for duplicates and removed them. The remaining articles were independently screened by two reviewers using titles and abstracts. The articles not excluded at this stage underwent full-text availability screening. Articles with full texts were screened against our selected eligibility criteria. Articles fulfilling our inclusion criteria were included in the meta-analysis. Any dispute regarding the selection of any article was solved with the help of a third reviewer. Additionally, one reviewer checked the references to identify any additional relevant articles.

The included articles were extracted by two reviewers. Data were extracted on author names, year of publication, trial numbers, sample size, male-to-female ratio, outcomes, intervention group, and the number of participants in that group.

Quality assessment and data synthesis

The potential for bias in the included RCTs was evaluated using the Risk of Bias 2.0 tool by The Cochrane Library [13]. It assigns one of the three main risk categories to articles, i.e., high risk, some concerns, or low risk. High-risk studies are those that show concerns in at least one domain or high risk in any domain. Studies with some concerns are those that have all domains with low risk, but one domain with some misgivings. Similarly, low-risk studies are those with minimal risk of bias across all five domains of the ROB2 tool.

RevMan was used to analyse the collected data. Mean differences were calculated and used for continuous outcomes in our study; a random-effects inverse-variance statistical model was used to compare the mean differences. I² was used as the measure of statistical heterogeneity, with heterogeneity above 75% considered high, <25% considered low, and 26-50% and 51%-75% considered mild and moderate, respectively. The meta-analysis was presented as forest plots, with a p-value of 0.05 for all outcomes. Sensitivity analysis was also conducted to rule out possible causes of heterogeneity. Subgroup analysis was initially planned, but couldn't be performed due to limited data availability.

Ethical considerations

This study synthesised data from previously published research and did not involve direct patient recruitment or the collection of individual patient data. Therefore, ethical approval and informed consent were not required.

Results

**Figure 1:**
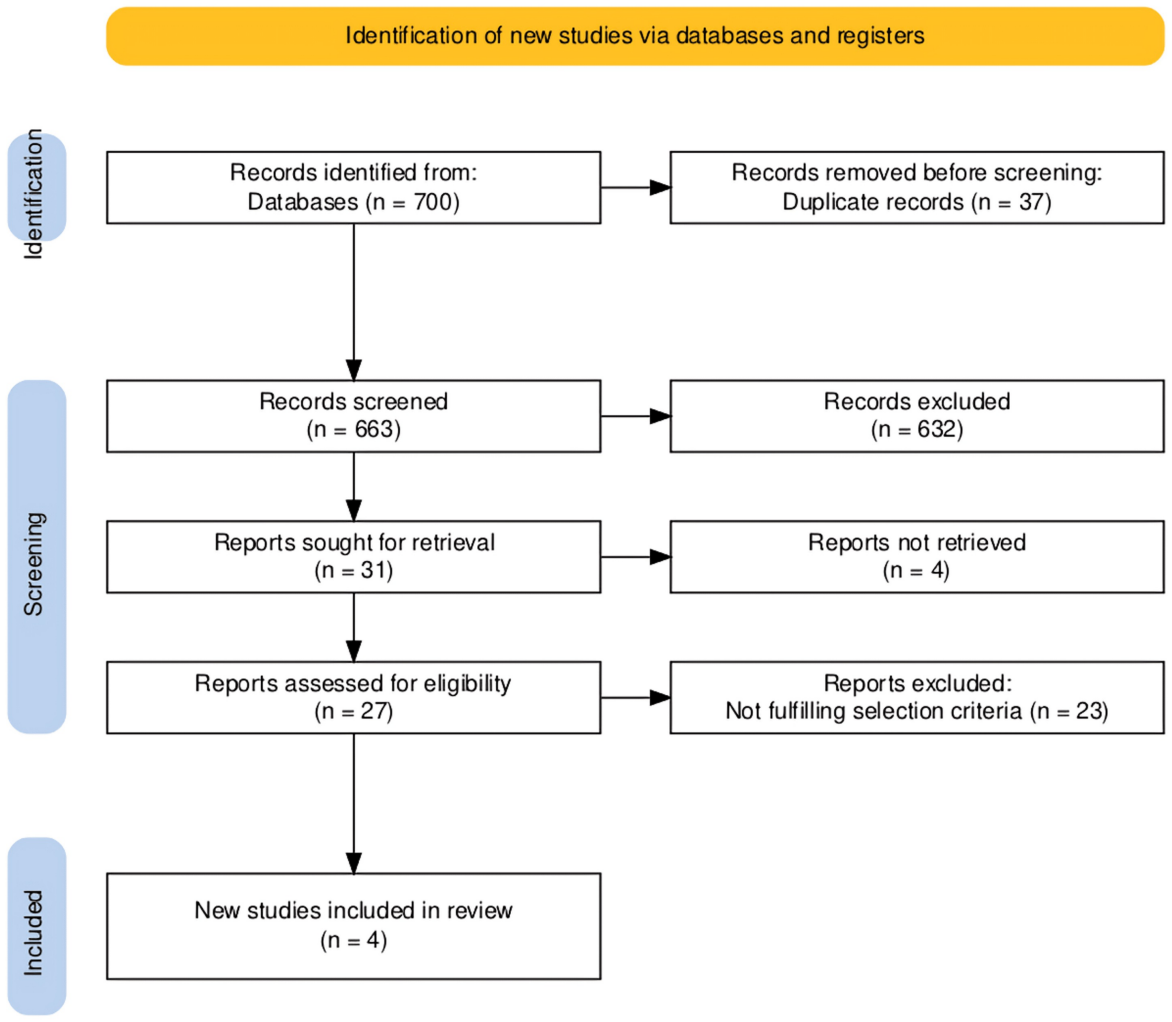
PRISMA flowchart for screening and selection of articles Flow diagram summarising study identification, screening, eligibility, and inclusion according to PRISMA guidelines.

We initially retrieved 700 articles from our included databases using specific keywords. During duplicate screening, we removed 37 articles. During title and abstract screening, we removed 632 articles, leaving only 31. The full text was searched for 31 articles, and only four were not retrieved. 27 articles underwent selection, leaving four, as 23 didn't meet our inclusion criteria and were excluded. Additionally, we could not find any relevant articles that met our eligibility criteria reported in the reference lists of the selected articles. The PRISMA diagram for screening and selection is shown in Figure 1.

Characteristics of studies

Our meta-analysis comprised four trials and 295 participants, of whom 150 were in the TNFi group and 145 in the control group. The majority of the population was female, i.e., 202 females and 93 males. Two studies reported age as the mean, while two reported it as the median. Three studies reported on BMI, DAS28-CRP, and RA disease duration: Tam LS et al., 2012, did not report BMI or DAS28-CRP, while O'Neill F et al., 2016, did not report RA disease duration. Two of the trials used infliximab, while two trials used adalimumab or etanercept as TNFi. Biomarkers for CV risk were observed across all trials. Table 1 summarises characteristics of the included studies.

**Table 1:** Characteristics of included studies Baseline demographic and clinical characteristics of included randomised controlled trials. Continuous variables are reported as mean ± standard deviation or median (interquartile range). TNFi indicates tumour necrosis factor inhibitor; BMI, body mass index; DAS28-CRP, Disease Activity Score using C-reactive protein [14-17].

-	Included Studies			
-	Tam LS et al., 2012 [14]	Solomon HD et al., 2022 [15]	O'Neill F et al., 2016 [16]	Liao KP et al., 2024 [17]
Sample Size	40	115	18	122
Age	Intervention: 53.0 (43.0, 59.0) Control: 53.0 (43.0, 59.0)	Intervention: 58.0 (53.0, 66.0) Control: 59.0 (54.0, 63.0)	Intervention: 61.30 (11.05) Control: 55.86 (15.89)	Intervention: 56.0 (53.0, 62.0) Control: 58.0 (54.0, 63.0)
Male: Female	5: 15	33: 82	6: 12	29: 93
BMI	-	Control: 28.8 (25.7, 32.9) Intervention: 29.5 (26.2, 34.1)	Control: 25.88 (0.55) Intervention: 24.98 (5.11)	Control: 29.6 (26.5, 34.7) Intervention: 29.7 (26.2, 34.5)
Hypertension	4 (20%) = placebo 6 (30%) = experimental	52 (45.2%)	Reported baseline blood pressures	47 (38.5)
Hyperlipidemia	0 (0%) = placebo 0 (0%) = experimental	23 (20%)	Reported baseline lipid panel	9 (7.4)
Diabetes Mellitus	0 (0%) = placebo 1 (5%) = experimental	2 (1.7%)	Reported baseline glucose levels	1 (0.8)
RA Disease Duration	Control: 5.8 (2.0, 11.9) Intervention: 4.2 (3.1, 8.6)	Control: 1.4 (0.5, 5.3) Intervention: 1.5 (0.5, 7.2)	-	Control: 1.4 (0.5, 5.3) Intervention: 2.5 (0.6, 7.2)
DAS28 CRP	-	Control: 4.6 (3.7, 5.6) Intervention: 4.9 (4.0, 5.5)	Control: 6.08 (0.71) Intervention: 5.17 (1.13)	Control: 4.8 (4.0, 5.7) Intervention: 5.0 (4.0, 5.5)
Intervention	TNFi (Infliximab 3 mg/kg at Weeks 0, 2, 6, and every 8 weeks) thereafter	TNFi (adalimumab 40 mg every other week or etanercept 50 mg every week)	TNFi (Infliximab 5 mg/kg at weeks 0, 2 and 6, and then every 8 weeks)	TNFi (adalimumab 40 mg every other week or etanercept 50 mg every week)
Outcomes	Biomarkers of CV risk, markers of inflammation	TBR in MDS, DAS28, CRP, Biomarkers of CV risk	Biomarkers of CV risk, HDL function, and RA disease activity	Biomarkers of CV risk, lipoproteins

Risk of bias

**Figure 2:**
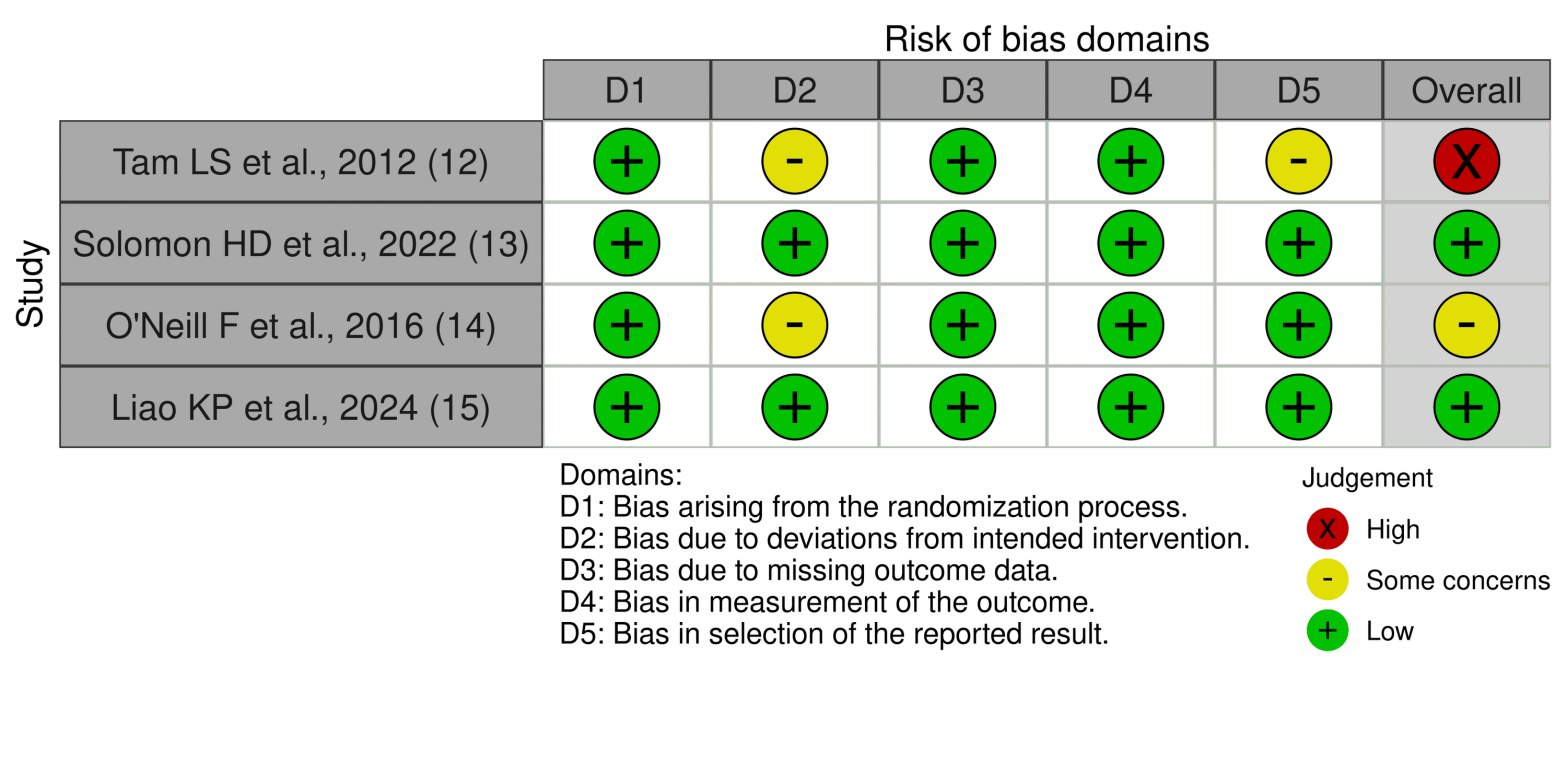
Summary of risk of bias Risk of bias assessment of included randomised controlled trials using the Cochrane Risk of Bias 2.0 tool across predefined domains. Included Studies: [14-17]

Risk of bias was assessed using ROB2, which showed that two studies had low risk, one had some concerns, and one had high risk. For the study, there were some concerns about the domain assessment bias in the deviation of the intervention. Tam LS 2012 et al. had a high risk of bias due to deviations from the intended intervention and the reported results domain. Figure 2 summarises the risk of bias for the included studies.

Effect on lipid profile

For LDL-C and total cholesterol, 4 RCTs reported means and standard deviations, whereas for triglycerides and HDL-C, only three trials reported means and SDs. Our analysis showed that reductions in LDL-C were greater in the control group than in the TNF-i group; although analysed, the difference did not reach significance (MD 0.35; 95% CI [-0.19, 0.88], p = .20), and the heterogeneity was low (I² = 12%). Similarly, for HDL-C, elevations in the control group were greater than in the TNF-i group; however, again, the results were not statistically significant, and mild statistical heterogeneity was observed (I² = 46%; MD = -0.85; 95% CI = [-3.15, 1.46], p = .47).

**Figure 3:**
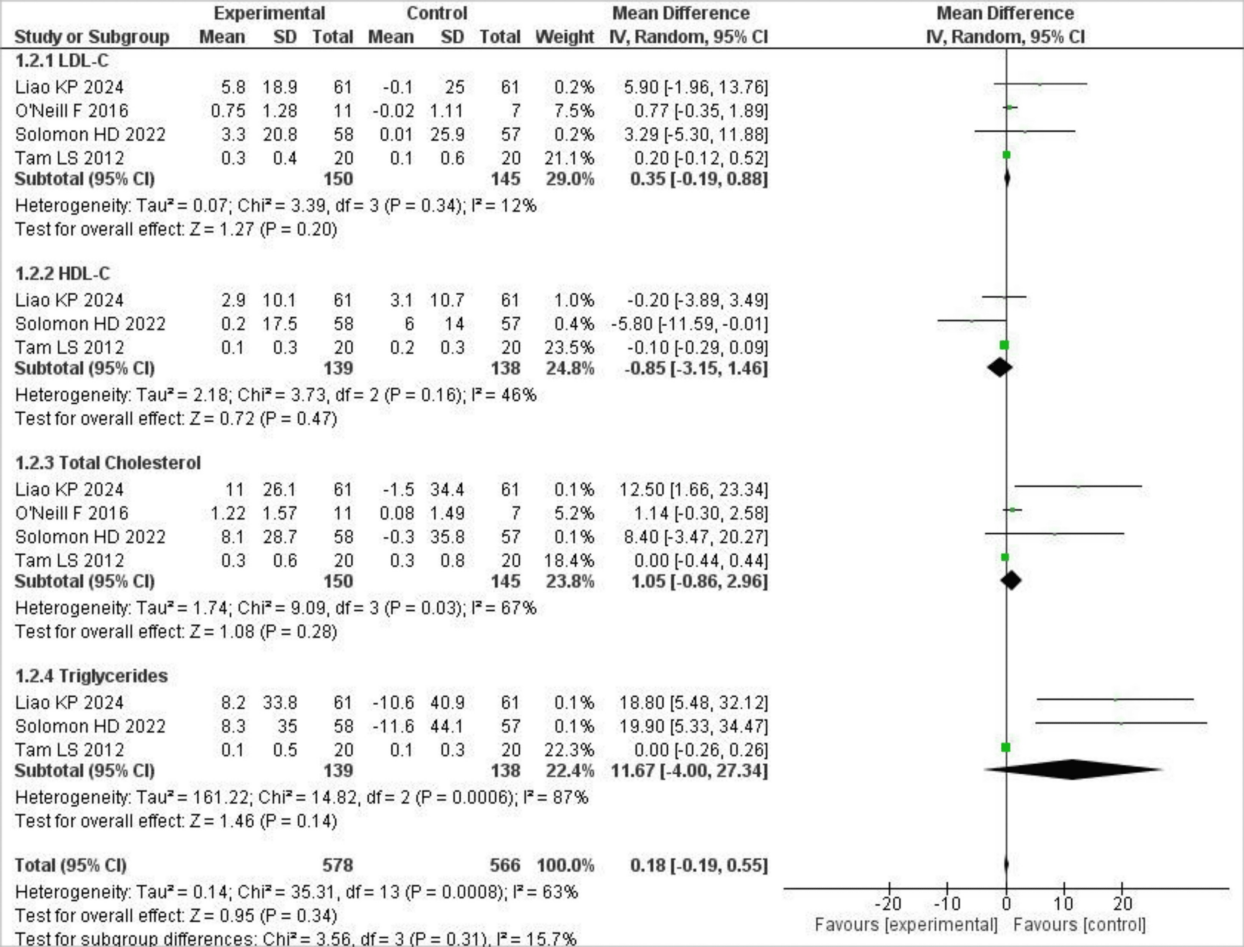
Forest plot showing lipid biomarkers of CV risk Forest plot showing pooled mean differences (MDs) with 95% confidence intervals (CIs) for LDL-C, HDL-C, triglycerides, and total cholesterol comparing anti-TNF therapy with control groups using a random-effects model. Heterogeneity was assessed using the I² statistic [14-17].

Results were not significant for triglycerides (MD 11.67; 95% CI [-4.00, 27.34], p = .14) and total cholesterol (MD 1.05; 95% CI [-0.86, 2.96], p = .28). However, the results were improved in the control group, although they remained insignificant. Additionally, statistical heterogeneity was high for the triglyceride outcome and moderate for the total cholesterol. The forest plot showing the above results for lipid markers is shown in Figure 3.

Effect on blood pressure

**Figure 4:**
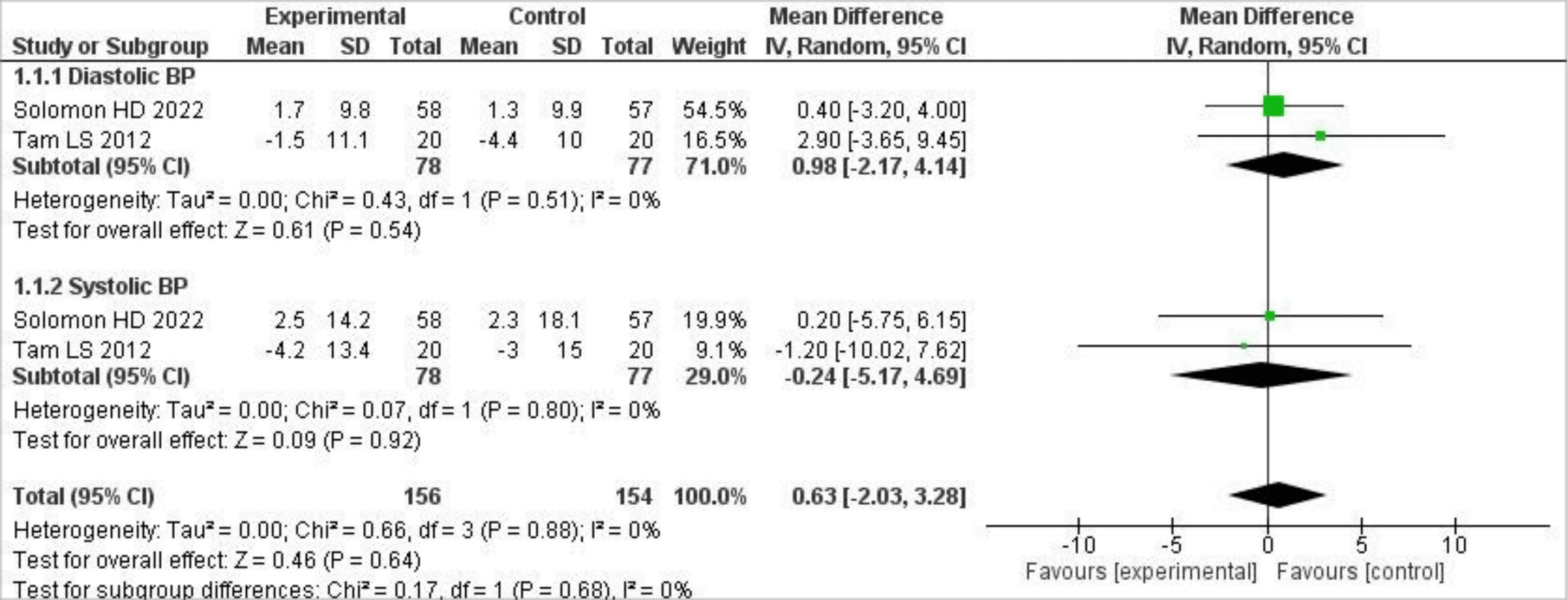
Forest plot showing the effect of TNFi on blood pressure compared to controls Forest plot presenting pooled mean differences (MDs) with 95% confidence intervals (CIs) for systolic and diastolic blood pressure comparing anti-TNF therapy with control interventions using a random-effects model [14,15].

Of the four included trials, only two reported effects on systolic and diastolic blood pressure. For SBP, TNF-i caused more reductions compared to controls, but the results were not significant (MD -24; 95% CI [-5.17, 4.69], p = .92). For DBP, more reductions were observed in the control group compared to the TNF-i group, but the findings didn’t reach statistical significance (MD 0.98; 95% CI [-2.17, 4.14], p = .54). No statistical heterogeneity was observed for both outcomes. The forest plot showing the results is given in Figure 4.

Sensitivity analysis

We conducted a leave-one-out analysis. Results for all outcomes did not differ significantly after sensitivity analysis; however, for triglycerides, results became significant in favour of the controls after removal of the study by Tam LS et al., 2012 (MD 19.30; 95% CI [9.47, 29.13], p = .0001) with no statistical heterogeneity observed.

Discussion

This systematic review and meta-analysis evaluated the effects of anti-TNF therapy on conventional cardiovascular risk-related biomarkers in patients with rheumatoid arthritis, specifically lipid parameters and blood pressure. Most included trials were judged to have a low or moderate risk of bias, while one study was rated as high risk due to deviations from the intended intervention. Such deviations may influence effect estimates and should be considered when interpreting pooled findings [18,19].

TNF-α inhibitors were not associated with statistically significant changes in lipid parameters in our pooled analysis; however, the limited number of RCTs, small sample sizes, and heterogeneity across studies make these findings inconclusive. Previous studies in rheumatoid arthritis and psoriasis reported variable or transient effects on lipid profiles, highlighting the uncertainty of the cardiovascular impact of TNF-α inhibitors [20-22]. This may reflect the complex interplay between systemic inflammation and lipid metabolism in RA, often referred to as the “lipid paradox.”

In our analysis, TNF-α inhibitor therapy was not associated with statistically significant changes in systolic or diastolic blood pressure compared with controls. These findings are consistent with previous RCTs and short-term monitoring studies, which reported minimal or variable effects of TNF inhibition on blood pressure [22-24]. However, since only a limited number of trials reported blood pressure outcomes, these results should be interpreted cautiously, and firm conclusions cannot be drawn regarding cardiovascular effects beyond lipid parameters.

Sensitivity analyses confirmed the robustness of most pooled estimates; however, the result for triglycerides was notably influenced by a single study, making the conclusion uncertain. This highlights the potential impact of individual trials on aggregated outcomes and underscores the need for larger, well-powered randomised controlled trials to provide more definitive evidence.

While previous meta-analyses have evaluated the cardiovascular effects of anti-TNF therapy in RA, they mostly focused on overall cardiovascular events or a mix of heterogeneous surrogate markers. By concentrating specifically on lipid profiles and blood pressure as practical surrogate indicators of cardiovascular risk, this systematic review synthesises the available RCT evidence up to 2025. This approach provides an updated, focused assessment of anti-TNF therapy’s potential impact on these surrogate outcomes, complementing prior evidence and identifying areas that require further investigation. Compared with earlier meta-analyses, our review offers a targeted evaluation of defined surrogate cardiovascular outcomes, emphasising the need for standardised reporting in future trials.

Limitations

This research has several limitations. To begin with, the meta-analysis involved four randomised controlled trials. The sample size was relatively small, limiting the statistical power of the analysis and increasing the risk of Type II error, and may limit its generalisability, as subgroup analysis could not be conducted due to limited data available. Second, differences in study populations, anti-TNFs used, comparators used, and follow-up and outcomes reported could have given a false impression of the actual treatment effects. Third, not every trial reported all outcomes of interest, especially blood pressure, which precludes a strong pooled evaluation of some cardiovascular outcomes. Fourth, a single study was rated at high risk of bias, which could have affected the pooled outcomes. Also, it was not possible to conduct subgroup and meta-regression analyses due to insufficient data. Fifth, the emphasis on traditional lipid parameters may be understated when considering possible cardiovascular advantages mediated by non-conventional mechanisms, including endothelial activity, arterial rigidity, or vascular inflammation. Finally, only studies published in English were included, potentially excluding relevant trials conducted in non-English-speaking populations, particularly in Asia. Future reviews should consider non-English studies or translated data to improve comprehensiveness and generalizability.

Future directions

Future research must focus on large, well-designed randomised controlled trials with longer follow-ups to better assess the cardiovascular impact of anti-TNF therapy in RA. There should be standardised reporting of cardiovascular events, both clinical and vascular surrogate markers. Direct comparative outcome studies of anti-TNF versus other biologic/targeted synthetic DMARDs could help further elucidate differences in cardiovascular effects. Further research on mechanistic pathways linking inflammation control to cardiovascular risk reduction, including advanced imaging and functional vascular evaluation, should also be considered. The addition of cardiovascular risk stratification to RA treatment algorithms could also be used to identify patient subgroups with the highest likelihood of responding to anti-TNF therapy.

## Conclusions

This systematic review and meta-analysis did not identify statistically significant differences between anti-TNF therapy and control treatments in conventional lipid profiles or blood pressure among patients with rheumatoid arthritis. While anti-TNF agents remain highly effective for controlling disease activity, their impact on cardiovascular risk markers remains uncertain when assessed using these surrogate measures. These findings underscore the need for larger, adequately powered randomised controlled trials with longer follow-up periods that evaluate not only conventional risk factors but also clinically relevant cardiovascular events and structural vascular surrogate markers to clarify the long-term cardiovascular effects of anti-TNF therapy in RA patients.
